# Brain trauma and family group therapy for acceptance and better communication

**DOI:** 10.1192/j.eurpsy.2023.2188

**Published:** 2023-07-19

**Authors:** D. Goujon

**Affiliations:** Secteur 1, Centre Hospitalier Spécialisé de l’Yonne, Sens, France

## Abstract

**Introduction:**

After severe brain trauma, patients undergo long periods of intrahospital treatment, rehabilitation and multidisciplinary evaluations. When they are sufficiently autonomous, they can be admitted to institution for health care, psychotherapy, occupational therapy as well as various efforts to improve their autonomy. The place taken by family can vary according to the project of the institution and their disponibility.

**Objectives:**

The family group therapy with an organized and structured program aim to improve the place that family have in this institution.

**Methods:**

Family group therapy can vary from support group to structured cognitive behavioural therapy and psycho-education. To meet our goal, we used the model from an experienced brain trauma center.

**Results:**

As a result, better communication between family and resident, family and staff, improved acceptance and a relief for families were found.

**Conclusions:**

In spite of lesser disponibility, the families already stress their need and gratitude for family group therapy.

**Disclosure of Interest:**

None Declared

